# A systematic review of treatment guidelines for metastatic colorectal cancer

**DOI:** 10.1111/j.1463-1318.2011.02765.x

**Published:** 2012-02

**Authors:** M S Edwards, S D Chadda, Z Zhao, B L Barber, D P Sykes

**Affiliations:** 1 PRMA Consulting Ltd, Centaur House, Ancells Business ParkHampshire, UK; 2 Global Health Economics, Amgen Inc., Thousand OaksCalifornia, USA

**Keywords:** Metastatic colorectal cancer, treatment guidelines, systematic review, monoclonal antibodies

## Abstract

**Aim:**

A systematic review of treatment guidelines for metastatic colorectal cancer (mCRC) was performed to assess recommendations for monoclonal antibody therapy in these guidelines.

**Method:**

Relevant papers were identified through electronic searches of MEDLINE, MEDLINE In Process, EMBASE and the Cochrane Library; through manual searches of reference lists; and by searching the Internet.

**Results:**

A total of 57 relevant guidelines were identified, 32 through electronic database searches and 25 through the website searches. The majority of guidelines were published between 2004 and 2010. The country publishing the most guidelines was the USA (12), followed by the UK (10), Canada (eight), France (eight), Germany (three), Australia (two), Spain (two) and Italy (one). In addition, eight European and three international guidelines were identified. As monoclonal antibody therapy for mCRC was not introduced until 2004, no firm recommendations for monoclonal antibody therapy were made in guidelines published between 2004 and 2006. Recommendations for monoclonal antibody therapy first appeared in 2007 and evolved as more data became available. The most recent international, European and US guidelines recommend combination chemotherapy with the addition of a monoclonal antibody for the first-line treatment of mCRC. Second-line treatment depends on the first-line regimen used. For chemoresistant mCRC, cetuximab or panitumumab are recommended as monotherapy in patients with wild-type *KRAS* tumours.

**Conclusion:**

The study indicates that recent treatment guidelines have recognized the role of monoclonal antibodies in the management of mCRC, and that treatment guidelines should be updated in a timely manner to reflect the most recently available data.

## Introduction

### Background

Colorectal cancer (CRC) is currently the third most common cancer worldwide [1]. Approximately 20–25% of patients with the disease already have metastases at the time of diagnosis and 50–60% of the remainder will develop metastases [2,3]. For most patients with metastatic CRC (mCRC), treatment is palliative rather than curative. The goals of systemic treatment in these patients are to prolong survival and to maintain quality of life for as long as possible. However, a small proportion of patients with mCRC (e.g. those whose metastases are confined to the liver) can be converted to a potentially curable state through surgical resection of the metastases after systemic therapy. For these patients, the goal of systemic treatment is to shrink the metastases [4].

A number of different drugs have significant antitumour activity in mCRC, including the systemic drugs 5-fluorouracil (5-FU), irinotecan, oxaliplatin, bevacizumab, cetuximab and panitumumab, and the oral drug capecitabine. Different combinations of these drugs, such as the FOLFOX regimen (leucovorin, 5-FU and oxaliplatin), the FOLFIRI regimen (leucovorin, 5-FU and irinotecan) and the XELOX regimen (oxaliplatin and capecitabine), with or without a monoclonal antibody agent, have been shown to improve outcomes in mCRC [5–11].

The monoclonal antibodies bevacizumab, cetuximab and panitumumab are more recent additions to the list of systemic drugs available for the treatment of mCRC. Bevacizumab, an antibody against vascular endothelial growth factor (VEGF), was first approved as a treatment for mCRC in 2004, followed by cetuximab (also in 2004) and panitumumab (2006). Cetuximab and panitumumab both target the epidermal growth factor receptor (EGFR) and are effective only in patients with wild-type *KRAS* mCRC [8,9,12]. Panitumumab is the only approved fully human anti-EGFR monoclonal antibody, while cetuximab is a chimeric antibody and bevacizumab is a humanized monoclonal antibody.

The current indications for monoclonal antibody therapy in mCRC differ in Europe and the USA and between the three monoclonal antibodies. Bevacizumab is indicated for the first- and second-line treatment of mCRC in combination with fluoropyrimidine-based chemotherapy. Cetuximab and panitumumab are indicated for wild-type *KRAS* mCRC as monotherapy, and cetuximab is also indicated in combination with chemotherapy in Europe and in combination with irinotecan in irinotecan-refractory wild-type *KRAS* mCRC in the USA (Table 1). However, the optimal use of these agents in the treatment of mCRC is still evolving as new data become available [10,11,13].

**Table 1:** Approved treatment regimens for monoclonal antibodies in mCRC.

Antibody	FDA-approved regimens	EMA-approved regimens
Bevacizumab	In combination with i.v. 5-FU-based chemotherapy for first- or second-line treatment	In combination with fluoropyrimidine-based chemotherapy
Cetuximab	As a single agent in EGFR-expressing mCRC after failure of both irinotecan- and oxaliplatin-based regimens or in patients who are intolerant to irinotecan-based regimens In combination with irinotecan in EGFR-expressing mCRC in patients who are refractory to irinotecan-based chemotherapy Not recommended for the treatment of mCRC with *KRAS* mutations in codons 12 or 13	In combination with chemotherapy or as a single agent in patients with EGFR-expressing, *KRAS* wild-type mCRC who have failed oxaliplatin- and irinotecan-based therapy and who are intolerant to irinotecan
Panitumumab	Single agent for EGFR-expressing mCRC with disease progression or following fluoropyrimidine, oxaliplatin and irinotecan chemotherapy regimens Not recommended for the treatment of mCRC with *KRAS* mutations in codons 12 or 13	Monotherapy in EGFR-expressing mCRC with non-mutated (wild-type) *KRAS* after failure of fluoropyrimidine-, oxaliplatin- and irinotecan-containing chemotherapy regimens

EGFR, epidermal growth factor receptor; EMA, European Medicines Agency; FDA, Food and Drug Administration; 5-FU, 5-fluorouracil; i.v., intravenous; *KRAS*, V-Ki-ras2 Kirsten rat sarcoma viral oncogene homolog; mCRC, metastatic colorectal cancer.

Sources: http://www.emea.europa.eu/, http://www.fda.gov/.

### Objectives

Many guidelines for the treatment of mCRC have been published. As new treatments for the disease become available, the complexity of treatment increases and it is therefore important that these guidelines provide appropriate guidance to clinicians for the treatment of mCRC. A systematic review was performed to identify treatment guidelines for mCRC and to assess the recommendations for monoclonal antibody therapy in these guidelines.

## Method

### Search strategy

The review question was to describe treatment guidelines for mCRC. The PICOS elements were as follows: participants, patients with mCRC; interventions, the search was divorced from interventions; comparisons, the search was divorced from comparisons; outcomes, treatment guidelines for mCRC; and study design, the search was divorced from study design and instead was based on disease state (mCRC) and treatment guidelines.

Relevant papers were identified through electronic searches of MEDLINE, MEDLINE In Process, the Excerpta Medica Database (EMBASE) and the Cochrane library. The searches were performed on 5 January 2010 and the search terms used are shown in Table 2. The MEDLINE and EMBASE searches were limited to papers published in the English language, whereas the Cochrane library search had no language restrictions. None of the searches were limited by date. Surveys, audits, editorials, letters to the editor, case reports or notes were excluded. In addition to electronic database searches, the reference lists of relevant studies were searched manually for further relevant studies. Searches of other web-based resources, including physician and surgical organizations, were also performed.

**Table 2:** Search terms used in the electronic database searches.

Metastatic colorectal cancer terms	
1	‘colorectal metastasis’: de
2	‘colon metastasis’: de
3	‘rectum metastasis’: de
4	(metasta^*^ AND {colorectal OR colon OR colonic)): ti
5	(metasta^*^ AND (rectum OR rectal OR rectocolonic)): ti
6	(metasta^*^ NEAR/6 (colorectal OR colon OR colonic)): ab
7	(metasta^*^ NEAR/6 (rectum OR rectal OR rectocolonic)): ab
8	mcrc: ti,ab
Colorectal cancer terms	
1	‘colon tumor’/exp
2	‘rectum tumor’/de
3	‘hereditary colorectal cancer syndrome’: de
4	‘non polyposis colorectal cancer’: de
5	‘dukes stage b colorectal cancer’: de
6	((colorectal OR colon OR colonic) NEXT/1 (adenoma OR adenomas)): ti,ab
7	((colorectal OR colonic) NEXT/1 (cancer OR carcinoma)): ti,ab
8	((colorectal OR colonic) NEXT/1 (neoplasia OR neoplasm OR neoplasms)): ti,ab
9	((colorectal OR colonic) NEXT/1 (tumor OR tumors)): ti,ab
10	((colorectal OR colonic) NEXT/1 (tumour OR tumours)): ti,ab
11	((rectum OR rectal OR rectocolonic) NEXT/1 (adenoma OR adenomas)): ti,ab
12	((rectum OR rectal OR rectocolonic) NEXT/1 (cancer OR carcinoma)): ti,ab
13	((rectum OR rectal OR rectocolonic) NEXT/1 (neoplasia OR neoplasm OR neoplasms)): ti,ab
14	((rectum OR rectal OR rectocolonic) NEXT/1 (tumor OR tumors)): ti,ab
15	((rectum OR rectal OR rectocolonic) NEXT/1 (tumour OR tumours)): ti,ab
Metastatic terms	
1	‘metastasis’/exp
2	‘advanced cancer’/de
3	‘cancer staging’/exp
4	(metasta^*^ OR advanced): ti,ab
Treatment guidelines terms	
1	‘practice guideline’/exp
2	standard/de
3	‘professional standard’/de
4	‘gold standard’/de
5	consensus/de
6	‘evidence based practice’/de
7	(guideline^*^ OR consensus): ti,ab
8	(‘best practice’ OR ‘best practices’): ti,ab
9	(‘clinical pathway’ OR ‘clinical pathways’): ti,ab
10	(clinical NEXT/2 (protocols OR protocol)): ti,ab

### Selection criteria

Citations/abstracts of identified studies were reviewed and assessed for relevance by two independent researchers. Full paper copies of studies considered to be relevant were then reassessed for inclusion against the criteria below. Disagreements between the two researchers, which were rare, were resolved by discussion until a consensus was reached.

Inclusion criteria for papers were global, national or regional treatment guidelines for mCRC from Australia, Canada, France, Germany, Italy, Spain, the UK or the USA. Exclusion criteria for guidelines were those published in countries not listed above; those for non-metastatic CRC; those on CRC prevention, screening, detection, diagnostics, mapping, staging, imaging, scanning, follow-up without treatment and/or prognostic/predictive factors; those evaluating neuroendocrine tumours; pathology-related guidelines; and unavailable papers.

### Data collection and analysis

Data from relevant publications were extracted into a data extraction table by three researchers, with the principal researcher overseeing all extraction. The following data were extracted: author, publication year, title, organizational body, country/region, publication type, language, target population, treatments included in guideline, and monoclonal antibody therapy guidance. Findings from the guidelines were summarized in tabular format. No statistical analyses were performed.

## Results

**Fig 1:**
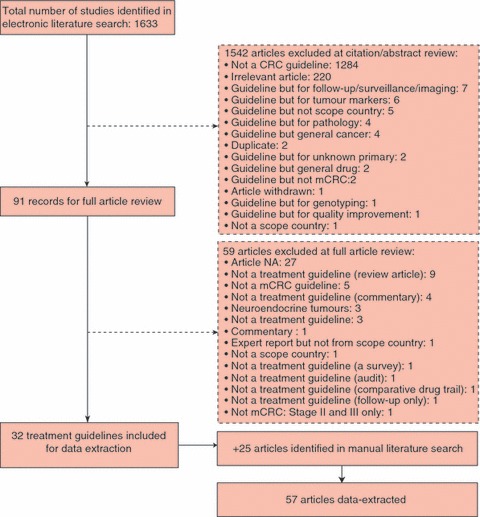
Flow diagram of the guideline selection process.

A total of 1633 citations/abstracts were identified in the initial searches. Of these, 1542 were excluded (reasons for exclusion are summarized in Fig. 1), leaving 91 for full-paper review. After analysis of the 91 full papers, 59 were excluded (reasons for exclusion are summarized in Fig. 1), leaving 32 relevant papers. In addition, 25 papers were identified from manual and website searches, giving a total of 57 relevant guidelines (see the Appendix).

**Fig 2:**
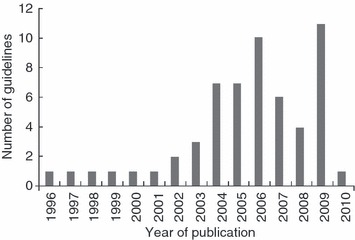
Summary of the number of published guidelines.

The 57 guidelines were published between 1996 and 2010, with the majority being published between 2004 and 2010 (Fig. 2). The country publishing the most guidelines was the USA (12), followed by the UK (10), Canada (eight), France (eight), Germany (three), Australia (two), Spain (two) and Italy (one). In addition, there were eight European guidelines [2,14–20] and three international guidelines [21–23].

### Timeframes

Of the 57 guidelines, only 11 were published prior to 2004 (the year in which monoclonal antibodies were introduced). The main treatment options discussed in these publications were surgery, chemotherapy and radiotherapy. As expected, none of the publications mentioned targeted therapy.

Twenty-four guidelines were published between 2004 and 2006. Over this period, the main treatment options discussed were again surgery, chemotherapy and radiotherapy. However, targeted therapy started to receive a mention, as did regional treatments, such as hepatic arterial infusion. No firm recommendations for targeted therapy were made apart from those in a consensus statement arising from a consensus conference sponsored by the American Hepato-Pancreato-Biliary Association (Table 3; Bartlett *et al.* (2006) [27]).

**Table 3:** Summary of findings from the international, European and US guidelines published since 2004.

Timeframe of publication	Region of publication	Organizational body	Authors and year	Treatments mentioned	Targeted therapy guidance
2004–2006	Europe	ESMO Guidelines Working Group (rectal cancer)	Tveit and Kataja 2005 [14]	Surgery Chemotherapy Radiotherapy	No mention of targeted therapy
ESMO Guidelines Working Group (colorectal cancer)	Van Cutsem *et al.* 2005 [15]	Surgery Chemotherapy Targeted therapy	moABs in combination with chemotherapy may be considered in carefully selected patients
European Colorectal Metastases Treatment Group	Van Cutsem *et al.* 2006 [18]	Surgery Chemotherapy HAI Targeted therapy	Not mentioned in consensus recommendations, but mentioned in future issues as the need to determine whether or not a combination of chemotherapy and targeted therapy will increase the number of patients eligible for resection
USA	Standards Practice Task Force of the American Society of Colon and Rectal Surgeons	Otchy *et al.* 2004 [24]	Surgery Chemotherapy Immunotherapy Radiotherapy	Very limited information: evidence from one study that postoperative treatment with monoclonal antibody 17-1A reduced overall mortality by 32% and recurrence by 23%
Society for Surgery of the Alimentary Tract (SSAT)	SSAT 2004 [25]	Surgery Chemotherapy HAI	No mention of targeted therapy
Consensus conference co-sponsored by American Hepato-Pancreato-Biliary Association, Society for Surgery of the Alimentary Tract, and Society of Surgical Oncology	Abdalla *et al.* 2006 [26]	Surgery Neoadjuvant chemotherapy Portal vein embolization Radiofrequency ablation	No mention of targeted therapy
Consensus conference sponsored by the American Hepato-Pancreato-Biliary Association	Bartlett *et al.* 2006 [27]	Chemotherapy Targeted therapy Regional hepatic therapy	For unresectable patients, the standard of care is FOLFIRI + bevacizumab or FOLFOX + bevacizumab If FOLFOX + bevacizumab is first-line therapy, irinotecan or FOLFIRI should be second-line; cetuximab should be added if progression occurs If FOLFIRI + bevacizumab is first-line therapy, FOLFOX or irinotecan + cetuximab should be second-line For resected patients, if bevacizumab is used as adjuvant therapy, the drug should be discontinued 8 weeks before surgery and/or wait 8 weeks following surgery
Society of Interventional Radiology Standards of Practice Committee	Brown *et al.* 2006 [28]	Regional hepatic therapy	No mention of targeted therapy
Consensus conference co-sponsored by American Hepato-Pancreato-Biliary Association, Society for Surgery of the Alimentary Tract, and Society of Surgical Oncology	Charnsangavej *et al.* 2006 [29]	Surgery	No mention of targeted therapy
2007–2011	International	International panel of 21 experts in colorectal oncology	Nordlinger *et al.* 2009 [22]	Surgery Chemotherapy Targeted therapy	Neoadjuvant chemotherapy can induce liver damage but there are few clinical consequences if patients are not overtreated; bevacizumab treatment is manageable in this setting provided proper care is taken Combination chemotherapy + bevacizumab or cetuximab can render initially unresectable metastases resectable in patients with advanced CRC; when choosing treatment consider the need for a delay between the end of bevacizumab treatment and surgery, and the fact that the response to cetuximab, like panitumumab, is limited to patients with wild-type *KRAS* tumours FOLFIRI or FOLFOX/XELOX + cetuximab could be a valuable first-line therapy for patients with wild-type *KRAS* tumours; same chemotherapy + bevacizumab could be valuable for unselected patients with mutated *KRAS* tumours
Task force of the International Society of Geriatric Oncology	Papamichael *et al.* 2009 [23]	Surgery Radiotherapy Chemotherapy Targeted therapy	Combination chemotherapy with or without bevacizumab should be the treatment of choice; cetuximab and panitumumab should be used within the context of their licensed indication Although data for the use of moABs in the treatment of elderly patients are lacking, it is unlikely that they have a different tolerance in the elderly than younger patients Panitumumab prolongs PFS in previously treated mCRC patients but should only be used in patients with wild-type KRAS tumours
Europe	European Colorectal Metastases Treatment Group	Nordlinger *et al.* 2007 [16]	Surgery Neoadjuvant chemotherapy Targeted therapy	Currently, the only targeted agent approved for first-line treatment of mCRC is bevacizumab, but it is not associated with high resectability and there are concerns about potential risks during surgery; hence, patients should stop therapy at least 6–8 weeks before liver resection
ESMO Guidelines Working Group	Glimelius and Oliveira 2008 [17]	Surgery Radiotherapy Chemotherapy Targeted therapy	First-line palliative chemotherapy with 5-FU/LV in various combinations and schedules with oxaliplatin or irinotecan with or without bevacizumab should be considered early
ESMO Guidelines Working Group	Van Cutsem and Oliveira 2008 [20]	Surgery Chemotherapy Targeted therapy	Chemotherapy + moABs should be considered in selected patients Bevacizumab increases OS and PFS in combination with an irinotecan-based regimen and PFS in combination with fluoropyrimidine + oxaliplatin as first-line therapy Cetuximab and panitumumab are active as single agents in chemo-refractory mCRC; cetuximab + irinotecan is more active than cetuximab monotherapy
ESMO Guidelines Working Group	Glimelius and Oliveira 2009 [19]	Surgery Radiotherapy Chemotherapy Targeted therapy	First-line palliative chemotherapy, consisting of 5-FU/LV in various combinations and schedules with oxaliplatin or irinotecan, with or without bevacizumab or cetuximab, should be considered early in patients with non-mutated *KRAS* tumours
ESMO Guidelines Working Group	Van Cutsem and Oliveira 2009 [2]	Surgery Chemotherapy Targeted therapy	Two cytotoxics + bevacizumab or cetuximab increases the resection rate of initially unresectable liver metastases Bevacizumab should be considered as it increases OS and PFS when given in various combinations as first- and second-line treatment Cetuximab and panitumumab are active as single agents in chemoresistant mCRC but their activity is confined to patients with *KRAS* wild-type tumours Cetuximab + irinotecan has become the reference treatment in chemoresistant *KRAS* wild-type tumours for patients who can tolerate the combination The activity of FOLFIRI is increased in first-line treatment when cetuximab is combined with FOLFIRI and FOLFOX in *KRAS* wild-type patients
USA	Expert panel on radiation oncology: rectal/anal cancer	Herman *et al.* 2007 [30]	Surgery Radiotherapy Chemotherapy Targeted therapy	New targeted therapies such as bevacizumab, cetuximab and panitumumab have increased the options available for treating mCRC
22 experts in colorectal cancer	Esquivel *et al.* 2008 [31]	Surgery Chemotherapy	No mention of targeted therapy
Society of Interventional Radiology Standards of Practice Committee	Brown *et al.* 2009 [32]	Regional hepatic therapy	No mention of targeted therapy
National Comprehensive Cancer Network	Engstrom *et al.* 2011 [33,34]	Surgery Chemotherapy Targeted therapy Radiotherapy Radiofrequency ablation	Current management of mCRC involves the use of various drugs either in combination or as single agents: 5-FU/LV, capecitabine, irinotecan, oxaliplatin, bevacizumab, cetuximab and panitumumab Initial therapy should be FOLFOX, CapeOX, FOLFIRI, 5-FU/LV or FOLFOXIRI; bevacizumab, cetuximab or panitumumab can be added to FOLFIRI or FOLFOX; cetuximab can also be added or CapeOX, 5-FU/LV or capecitabine Following first progression in wild-type *KRAS* mCRC: FOLFIRI + cetuximab or panitumumab; cetuximab plus irinotecan; or single-agent cetuximab or panitumumab After second progression in wild-type *KRAS* mCRC: cetuximab plus irinotecan, or single-agent cetuximab or panitumumab If bevacizumab, cetuximab or panitumumab are used for first-line therapy, they are not recommended for second or subsequent lines of therapy If bevacizumab is not used for initial therapy, it can be used following disease progression Chemotherapy + bevacizumab is recommended as neoadjuvant therapy for patients with resectable CRC with metastases in the liver and lung only

CapeOX, capecitabine + oxaliplatin; ESMO, European Society for Medical Oncology; FOLFIRI, LV, 5-FU and irinotecan; FOLFOX, LV, 5-FU and oxaliplatin; FOLFOXIRI, FOLFIRI + oxaliplatin; 5-FU, 5-fluorouracil; HAI, hepatic arterial infusion; LV, leucovorin; mCRC, metastatic colorectal cancer; moABs, monoclonal antibodies; OS, overall survival; PFS, progression-free survival; XELOX, oxaliplatin + capecitabine.

A total of 22 guidelines were published over the period 2007–2010. Recommendations for targeted therapy appeared in 16 of these guidelines. Recommendations from the international, European and US guidelines published from 2004 onwards are summarized in Table 3 (country specific guidelines other than the US are not presented here).

### Line of therapy

Overall (2007–2010), 16 guidelines gave guidance on or discussed targeted therapies. Of these, seven included guidance or discussion on all three approved monoclonal antibodies [20,22,23,30, 33–35] and six included either bevacizumab only, or cetuximab only, or bevacizumab and cetuximab [16,17, 36–39].

When the recommendations for targeted therapy from international, European and US guidelines were grouped according to line of therapy and date of publication, a clear pattern was seen (Table 4). For first-line therapy, recommendations started in 2006 for combination chemotherapy plus bevacizumab. Cetuximab did not appear in recommendations until 2009, when it was recommended for use in patients with wild-type *KRAS* tumours.

**Table 4:** Summary of recommendations for targeted therapy from international, European and US guidelines according to line of therapy.

Line of therapy	Authors and year	Organizational body	Recommendations
First-line therapy	Bartlett *et al.* 2006 [27]	Consensus conference sponsored by the American Hepato-Pancreato-Biliary Association	FOLFOX or FOLFIRI + bevacizumab
Nordlinger *et al.* 2007 [16]	European Colorectal Metastases Treatment Group	5-FU-based chemotherapy + bevacizumab
Glimelius and Oliveira 2008 [17]	ESMO Guidelines Working Group	5-FU/LV + oxaliplatin or irinotecan ± bevacizumab
Van Cutsem and Oliveira 2008 [20]	ESMO Guidelines Working Group	Fluoropyrimidine + oxaliplatin + bevacizumab or Irinotecan-based regimen + bevacizumab
Nordlinger *et al.* 2009 [22]	International panel of 21 experts in colorectal oncology	FOLFOX or FOLFIRI + bevacizumab in unselected patients with mutated *KRAS* tumours FOLFOX or FOLFIRI + cetuximab in patients with wild-type *KRAS* tumours
Papamichael *et al.* 2009 [23]	Task force of the International Society of Geriatric Oncology	Combination chemotherapy ± bevacizumab Cetuximab and panitumumab should be used within their licensed indications
Glimelius and Oliveira 2009 [19]	ESMO Guidelines Working Group	5-FU/LV + oxaliplatin or irinotecan ± bevacizumab or cetuximab in patients with non-mutated *KRAS* tumours
Van Cutsem and Oliveira 2009 [2]	ESMO Guidelines Working Group	Combination chemotherapy + bevacizumab FOLFOX or FOLFIRI + cetuximab in patients with *KRAS* wild-type tumours
Engstrom *et al.* 2011 [33,34]	US National Comprehensive Cancer Network	FOLFOX, FOLFIRI, CapeOx, 5-FU/LV, FOLFOXIRI or capecitabine Bevacizumab, cetuximab or panitumumab can be added to FOLFOX or FOLFIRI Bevacizumab can also be added to CapeOx, 5-FU/LV or capecitabine
Second-line therapy	Bartlett *et al.* 2006 [27]	Consensus conference sponsored by the American Hepato-Pancreato-Biliary Association	FOLFOX, FOLFIRI or irinotecan + cetuximab if FOLFOX or FOLFIRI + bevacizumab used for first-line therapy
Van Cutsem and Oliveira 2009 [2]	ESMO Guidelines Working Group	Combination chemotherapy + bevacizumab
Engstrom *et al.* 2011 [33,34]	US National Comprehensive Cancer Network	Following first progression in wild-type *KRAS* mCRC: FOLFIRI + cetuximab or panitumumab; cetuximab plus irinotecan; or single-agent cetuximab or panitumumab After second progression in wild-type *KRAS* mCRC: cetuximab plus irinotecan, or single-agent cetuximab or panitumumab If bevacizumab, cetuximab or panitumumab are used for first-line therapy, they are not recommended for second or subsequent lines of therapy If bevacizumab is not used for initial therapy, it can be used following disease progression
Chemoresistant mCRC	Van Cutsem and Oliveira 2008 [20]	ESMO Guidelines Working Group	Cetuximab or panitumumab as single agents Irinotecan + cetuximab
Van Cutsem and Oliveira 2009 [2]	ESMO Guidelines Working Group	Cetuximab or panitumumab as single agents in patients with wild-type *KRAS* tumours Irinotecan + cetuximab in patients with wild-type *KRAS* tumours

CapeOX, capecitabine + oxaliplatin; ESMO, European Society for Medical Oncology; FOLFIRI, LV, 5-FU and irinotecan; FOLFOX, LV, 5-FU and oxaliplatin; FOLFOXIRI, FOLFIRI + oxaliplatin; 5-FU, 5-fluorouracil; LV, leucovorin; mCRC, metastatic colorectal cancer.

The most recent European guidelines from the European Society of Medical Oncology (ESMO) recognized the roles of bevacizumab and cetuximab (wild-type *KRAS* mCRC only) in the first-line treatment of mCRC, as well as cetuximab and panitumumab monotherapy in patients with chemo-refractory and wild-type *KRAS* mCRC (Table 4; Glimelius and Oliveira 2009 [19], Van Cutsem and Oliveira 2009 [2]). The most recent US guidelines from the National Comprehensive Cancer Network [33,34] recognized the role of all three monoclonal antibodies in the early-line treatment of mCRC. These guidelines recommended that initial therapy for mCRC should consist of FOLFOX, CapeOX, FOLFIRI, 5-FU/leucovorin, FOLFOXIRI or capecitabine, with bevacizumab, cetuximab or panitumumab being added to FOLFIRI or FOLFOX (wild-type *KRAS* mCRC only for cetuximab and panitumumab), and bevacizumab being added to CapeOX, 5-FU/leucovorin or capecitabine.

For second-line therapy, the guidelines from the US National Comprehensive Cancer Network [33,34] stated that possible treatment options following first progression in patients with wild-type *KRAS* mCRC were FOLFIRI plus cetuximab or panitumumab; cetuximab plus irinotecan; or single-agent cetuximab or panitumumab. Treatment options after second progression in patients with wild-type *KRAS* mCRC included cetuximab plus irinotecan, or single-agent cetuximab or panitumumab. If bevacizumab was used in a first-line regimen, it was not recommended for second or subsequent lines of therapy. Similarly, if cetuximab or panitumumab were used as part of the initial treatment regimen, neither agent was recommended in second or subsequent lines of therapy.

For chemoresistant mCRC, European guidelines also recognized the effectiveness of cetuximab or panitumumab as single agents, or irinotecan plus cetuximab, in patients with wild-type *KRAS* tumours [2].

### Specific patient populations

The two most recent international treatment guidelines addressed specific patient populations. The guidelines from an international panel of 21 colorectal oncology experts [22] focused on patients with colorectal liver metastases, and stated that FOLFIRI, FOLFOX or XELOX with the addition of cetuximab was a valuable first-line therapy in patients with wild-type *KRAS* mCRC, while FOLFIRI, FOLFOX or XELOX with bevacizumab was valuable in unselected patients.

The task force of the International Society of Geriatric Oncology [23] developed guidelines for the treatment of elderly colorectal cancer patients. It recommended that combination chemotherapy with or without bevacizumab should be the treatment of choice; cetuximab and panitumumab should be used within the context of their licensed indications in patients with wild-type *KRAS* mCRC. These guidelines also considered safety in the elderly patient population, stating that monoclonal antibodies are generally safe; however, bevacizumab, in particular, has a side-effect profile that includes hypertension (the most frequent side-effect), proteinuria, thromboembolic events, bleeding, wound healing complications and bowel perforation. These possible side-effects require careful consideration when treating elderly CRC patients. In particular, arterial thromboembolic events following bevacizumab were more likely to occur in patients over 65 years of age or in those who had a previous history (> 18%) of such events [23].

### Other guidelines

National Institute for Health and Clinical Excellence (NICE) in the UK published guidance on cetuximab for the first-line treatment of mCRC [39]. This single-technology appraisal recognized the role of cetuximab in the treatment of mCRC for a confined sub-patient population. The guidelines recommended cetuximab in combination with FOLFOX (or FOLFIRI in patients who were unable to tolerate or had contraindications to oxaliplatin), within its licensed indication, only when all of the following criteria were met: the primary colorectal tumour had been resected or was potentially operable; the metastatic disease was confined to the liver and was unresectable; and the patient was fit enough to undergo surgery to resect the primary colorectal tumour and to undergo liver surgery if the metastases became resectable after treatment with cetuximab.

The most recent Canadian guidelines [35] recommended that patients with mCRC and an Eastern Cooperative Oncology Group (ECOG) performance status of 0, 1 or 2 should be offered palliative chemotherapy. Whether treatment was with combination chemotherapy or sequential monotherapy (with or without bevacizumab) depended upon the patient’s treatment goals, their physical status and other life circumstances, as assessed by the treating oncologist. The guidelines recognized that cetuximab and panitumumab delay disease progression compared with best supportive care in patients with *KRAS* wild-type mCRC who are refractory or intolerant to fluoropyrimidine, irinotecan and oxaliplatin. However, no firm recommendations for their use were made.

According to recent international, European and US treatment guidelines, the generally agreed recommendations for chemotherapy regimens in the early-line treatment of mCRC are for 5-FU/leucovorin in various combinations and schedules with oxaliplatin or irinotecan, with all guidelines recommending FOLFOX and FOLFIRI and a few also including XELOX [2,22,23, 33,34,39].

## Discussion

The results of this systematic review show that recent international, European and US treatment guidelines for mCRC have recognized the role of and recommended use of monoclonal antibodies in the management of this disease. It is also noted that treatment guidelines, particularly those specific to individual countries, are outdated, as they do not reflect the most recently available data.

As expected, no firm recommendations for monoclonal antibody therapies were made over the period 2004–2006, a time when bevacizumab, cetuximab and panitumumab had just been introduced for the treatment of mCRC. Recommendations for the use of monoclonal antibody therapy did not appear in guidelines until around 2007. The guidelines started with recommendations for the addition of bevacizumab to combination chemotherapy for the first-line treatment of mCRC, and evolved into recommendations for the use of all three monoclonal antibodies as new data became available.

The most recent ESMO treatment guidelines published in 2009 [2] more closely reflect the European Medicines Agency approved indications for the three monoclonal antibodies in mCRC, as their recommendations include bevacizumab in combination with chemotherapy in first- and second-line therapy; cetuximab combined with chemotherapy as early-line treatment for patients with wild-type *KRAS* mCRC; and both cetuximab and panitumumab as monotherapy in chemo-refractory wild-type *KRAS* mCRC.

By contrast, the US National Comprehensive Cancer Network treatment guidelines [33,34] recognize the most recently available clinical data for cetuximab and panitumumab in combination with standard chemotherapy in the first-line and second-line treatment of wild-type *KRAS* mCRC, even though in the USA cetuximab is only indicated either in combination with irinotecan for irinotecan-refractory patients or as monotherapy for chemo-refractory patients, and panitumumab is only approved as a single agent in chemo-refractory mCRC.

For example, in the phase III PRIME trial, panitumumab in combination with FOLFOX4 significantly improved progression-free survival compared with FOLFOX4 alone in the first-line treatment of *KRAS* wild-type mCRC [10]. Another phase III trial demonstrated that panitumumab in combination with FOLFIRI significantly improved progression-free survival compared with FOLFIRI alone in the second-line treatment of wild-type *KRAS* mCRC [11]. Furthermore, panitumumab in combination with chemotherapy has also consistently demonstrated a trend in overall survival improvement, although not statistically significant, in both the first-line and second-line treatment of patients with wild-type *KRAS* mCRC.

Similarly, a recent trial of first-line treatment with cetuximab in combination with FOLFIRI showed that such treatment reduced the risk of disease progression compared with FOLFIRI alone in patients with *KRAS* wild-type tumours [8]. Another trial of first-line treatment in patients with wild-type *KRAS* mCRC showed that a combination of cetuximab and FOLFOX4 increased the likelihood of a response and was associated with a lower risk of disease progression than treatment with FOLFOX4 alone [40].

It is also worth noting that the recent international, European and US treatment guidelines [2,22,23,33,34] recommend FOLFOX and FOLFIRI, with some also including XELOX, as the main chemotherapy choice or as the chemotherapy backbones for combining with monoclonal antibodies in the early-line treatment of mCRC. Finally, these guidelines discourage using the same monoclonal antibody in subsequent lines of therapy. Specifically, if bevacizumab is used in a first-line regimen, it is not recommended for second or subsequent lines of therapy; similarly, if cetuximab or panitumumab is used as part of the initial treatment regimen, neither agent is recommended for second or subsequent lines of therapy.

A large proportion of the guidelines identified in this review were retrieved by manual searching, including a search of grey literature on the Internet (25/57; 44%). Possible reasons for the manual identification of such a large proportion of relevant papers include the fact that the searching of electronic databases relies on the correct indexing of papers and the use of appropriate key words in the titles/abstracts of papers. Since guidelines, expert consensus statements and recommendations do not always fulfil these criteria, they can prove difficult to retrieve from an electronic search. In addition, organizations often publish guidelines on the Internet rather than in journals, so the guidelines can only be retrieved through grey literature searches.

A limitation of this review is the fact that the content of non-English language guidelines was not assessed, apart from those identified in the Cochrane library. Thus, it was not possible to assess whether or not such guidelines reflect the most recent data. However, it is likely that non-English language treatment guidelines were specific to individual countries. The review was restricted to guidelines from eight countries only, another limitation. In future the review can expand to include Asia and other European countries. Finally the scientific validity and methodological quality of the included guidelines was not assessed. This was beyond the scope defined for this review, particularly as the aim was identification of the relevant guidelines rather than quality assessment of the guidelines; therefore the opportunity remains for this type of research in the future. There are various tools available for the quality assessment of guidelines such as the AGREE instrument [41] and the ADAPTE evaluation [42].

In conclusion, the findings from this study indicate that recent international, European and US treatment guidelines have recognized the role of monoclonal antibody agents in the management of mCRC. Since clinical data with monoclonal antibodies and other therapies in the treatment of mCRC are continually evolving, it is important that treatment guidelines are updated in a timely manner to reflect the most recently available clinical data.

## Appendix

Abad A, Figueras J, Valls C *et al.* Guidelines for the detection and treatment of liver metastases of colorectal cancer. *Clin Transl Oncol* 2007; **9**: 723–30.

Abdalla EK, Adam R, Bilchik AJ, Jaeck D, Vauthey JN, Mahvi D. Improving resectability of hepatic colorectal metastases: expert consensus statement. *Ann Surg Oncol* 2006; **13**: 1271–80.

Alberta Health Services Clinical Practice Guideline. *Metastatic Colorectal Cancer. 2009*. http://www.cancerboard.ab.ca/NR/rdonlyres/C2CB311E-D7B0-4D55-9318-499120A26579/0/GI003ColorectalMetastaticAug2009.pdf. Accessed February 2010

Anseline PF, Bell A, McK Bokey EL *et al.* Best practice parameters for management of rectal cancer: Recommendations of the Colorectal Surgical Society of Australia. *ANZ J Surg* 1996; **66**: 508–14.

Association for Coloproctology of Great Britain and Ireland (2007, 3rd edition). *Guidelines for the Management of Colorectal Cancer*. http://www.acpgbi.org.uk/assets/documents/COLO_guides.pdf. Accessed February 2010

Australian Cancer Network Colorectal Cancer Guidelines Revision Committee. *Clinical Practice Guidelines for the Prevention, Early Detection and Management of Colorectal Cancer. The Cancer Council Australia and Australian Cancer Network, 2005.*http://www.nhmrc.gov.au/_files_nhmrc/file/publications/synopses/cp106/cp106.pdf. Accessed February 2010

Bartlett DL, Berlin J, Lauwers GY, Messersmith WA, Petrelli NJ, Venook AP. Chemotherapy and regional therapy of hepatic colorectal metastases: expert consensus statement. *Ann Surg Oncol* 2006; **13**: 1284–92.

Bécouarn Y, Guillo S, Artru P *et al. Standards, options and recommendations. Intérêt de la chimiothérapie péri-opératoire dans la prise en charge des patients atteints d’un adénocarcinome du rectum résécable d’emblée (rapport intégral). L’Institut National du Cancer (INCa), 2007.*http://www.sor-cancer.fr/index.php?tg=articles&topics=65. Accessed February 2010

Bouche O. *Cancer du côlon métastatique. La Société Nationale Française de Gastroentérologie (SNFGE), 2009*. http://www.snfge.asso.fr/data/ModuleDocument/publication/5/pdf/TNCD-chapitre-960.pdf. Accessed February 2010

Brown DB, Cardella JF, Sacks D *et al.* Quality improvement guidelines for transhepatic arterial chemoembolization, embolization, and chemotherapeutic infusion for hepatic malignancy. *J Vasc Interv Radiol* 2006; **17**: 225–32.

Brown DB, Cardella JF, Sacks D *et al.* Quality improvement guidelines for transhepatic arterial chemoembolization, embolization, and chemotherapeutic infusion for hepatic malignancy. *J Vasc Interv Radiol* 2009; **20**(Suppl. 7): S219–26.

Charnsangavej C, Clary B, Fong Y, Grothey A, Pawlik TM, Choti MA. Selection of patients for resection of hepatic colorectal metastases: expert consensus statement. *Ann Surg Oncol* 2006; **13**: 1261–8.

Conroy T, Adenis A, Bouché O *et al. Standards, options and recommendations: recommandations pour la pratique clinique. Prise en charge par chimiothérapie palliative de première ligne des patients atteints d’un cancer colorectal métastatique. Bulletin de synthèse de veille. L’Institut National du Cancer (INCa), 2005.*http://www.sor-cancer.fr/index.php?tg=articles&topics=44. Accessed February 2010

Conroy T, Gory-Delabaere G, Adenis A *et al.* Clinical practice guideline: 2003 Update of standards, options and recommendations for first line palliative chemotherapy in patients with metastatic colorectal cancer (summary report). *Bull Cancer* 2004; **91**: 759–68.

Engstrom PF, Arnoletti JP, Benson AB 3rd *et al.* (2011, version 1.2011) *NCCN Clinical Practice Guidelines in Oncology: colon cancer.*http://www.nccn.org/professionals/physician_gls/f_guidelines.asp#site. Accessed February 2011

Engstrom PF, Arnoletti JP, Benson AB 3rd *et al.* (2011, version 1.2011) *NCCN Clinical Practice Guidelines in Oncology: rectal cancer.*http://www.nccn.org/professionals/physician_gls/f_guidelines.asp#site. Accessed February 2011

Esquivel J, Elias D, Baratti D, Kusamura S, Deraco M. Consensus statement on the loco regional treatment of colorectal cancer with peritoneal dissemination. *J Surg Oncol* 2008; **98**: 263–7.

Faivre J. Consensus conference. Prevention, diagnosis and treatment of colon cancer. *Gastroenterol Clin Biol* 1998; **22**: 205–26.

Fédération Francophone de Cancérologie Digestive (FFCD). What can be done for patients with cancer of the digestive tract in 2003? Guidelines of the francophone federation of digestive tract concerology – Part I. *Gastroenterol Clin Biol* 2004; **26**: 1140–64.

Figueredo A, Moore M, Germond C *et al.* Use of irinotecan in secondline treatment of metastatic colorectal carcinoma. *Curr Oncol* 2000; **7**: 29–36.

Garangou AC, Castillejo MM, Romero FJA *et al. Guía de práctica clinica: prevención del cáncer colorrectal. Work Group for the Clinical Practice Guidelines for the Prevention of Colon Cancer, 2009.*http://www.guiasgastro.net/guias_full/textos/ccolon.pdf. Accessed February 2010

Garden OJ, Rees M, Poston GJ *et al.* Guidelines for resection of colorectal cancer liver metastases. *Gut* 2006; **55**(Suppl. 3): iii1–8.

Germond C, Maroun J, Zwaal C *et al.* Use of raltitrexed in the management of metastatic colorectal cancer. *Curr Oncol* 1999; **6**: 217–23.

Glimelius B, Oliveira J. Rectal cancer: ESMO clinical recommendations for diagnosis, treatment and follow-up. *Ann Oncol* 2008; **19**(Suppl. 2): ii31–2.

Glimelius B, Oliveira J. Rectal cancer: ESMO clinical recommendations for diagnosis, treatment and follow-up. *Ann Oncol* 2009; **20**(Suppl. 4): iv54–6.

Graeven U, Schmoll HJ. Interdisciplinary guideline conference ‘Colorectal carcinoma: prevention, diagnosis and treatment 2004’: Subject group VII: therapeutic management in metastases and in the palliative situation. *Coloproctology* 2006; **28**: 59–63.

Herman J, Messersmith WA, Johnstone PA *et al.* Expert Panel on Radiation Oncology – Rectal/Anal Cancer. *ACR Appropriateness Criteria® rectal cancer – metastatic disease at presentation, 2007.) American College of Radiology (ACR), Reston (VA).*http://www.guideline.gov/summary/summary.aspx?doc_id=13620&nbr=006981&string=colorectal. Accessed January 2010

Italian National Institute of Health (ISS)/Regional Health Service Agency (ASSR). *National Guidelines: Colon-Rectal Cancer, 2002.*http://www.snlg-iss.it/lgn_cancro_colon_retto. Accessed February 2010

Jonker D, Rumble RB, Maroun J. Role of oxaliplatin combined with 5-fluorouracil and folinic acid in the first- and second-line treatment of advanced colorectal cancer. *Curr Oncol* 2006; **13**: 173–84.

Kavanagh M, Ouellet JF. Clinical practice guideline on peritoneal carcinomatosis treatment using surgical cytoreduction and hyperthermic intraoperative intraperitoneal chemotherapy. *Bull Cancer* 2006; **93**: 867–74.

Kocha W, Maroun J, Jonker D, Rumble RB, Zuraw L, and the Gastrointestinal Cancer Disease Site Group of Cancer Care Ontario’s Program in Evidence-based Care. Oral capecitabine (Xeloda) in the first-line treatment of metastatic colorectal cancer. *Curr Oncol* 2004; **11**: 81–92.

*Kocha W, Maroun J, Jonker D, Rumble RB, Zuraw L. Oral capecitabine (Xeloda) in the first-line treatment of metastatic colorectal cancer: a clinical practice guideline. (Evidence-based series; no. 2-15, 2005). Cancer Care Ontario (CCO), Toronto (ON).*http://www.cancercare.on.ca/common/pages/UserFile.aspx?fileId=13976. Accessed February 2010

Lazorthes F. Recommendations for clinical practice: choices of treatment for rectal cancer. November 2005. *Oncologie* 2006; **8**: 773–84.

Lazorthes F, Navarro F, Ychou M, Delpero JR, Rougier P. Therapeutic management of hepatic metastases from colorectal cancers. *Gastroenterol Clin Biol* 2003; **27** (Spec No. 2): B7.

Nelson H, Petrelli, N, Carlin A *et al.* Guidelines 2000 for colon and rectal cancer surgery. *J Natl Cancer Inst* 2001; **93**: 583–96.

NHS National Institute for Health and Clinical Excellence (NICE). Guidance on the use of capecitabine and tegafur with uracil for metastatic colorectal cancer. *Technology Appraisal Guidance 61, 2003*. http://www.nice.org.uk/nicemedia/pdf/61CapecitabineCRCfullguidance.pdf. Accessed February 2010

NHS National Institute for Health and Clinical Excellence (NICE). Improving outcomes in colorectal cancer: manual update. *Guidance on Cancer Services, 2004.*http://www.nice.org.uk/nicemedia/pdf/CSGCCfullguidance.pdf. Accessed February 2010

NHS National Institute for Health and Clinical Excellence (NICE). Selective internal radiation therapy for colorectal metastases in the liver. *Interventional Procedure Guidance 93, 2004.*http://www.nice.org.uk/nicemedia/pdf/ip/IPG093guidance.pdf. Accessed February 2010

NHS National Institute for Health and Clinical Excellence (NICE). Irinotecan, oxaliplatin and raltitrexed for the treatment of advanced colorectal cancer. *NICE Technology Appraisal Guidance 93, 2005.*http://www.nice.org.uk/nicemedia/pdf/TA93Guidance.pdf. Accessed February 2010

NHS National Institute for Health and Clinical Excellence (NICE). Bevacizumab and cetuximab for the treatment of metastatic colorectal cancer. *NICE Technology Appraisal Guidance 118, 2007*. http://www.nice.org.uk/nicemedia/pdf/TA118Guidance.pdf. Accessed February 2010

NHS National Institute for Health and Clinical Excellence (NICE). Cetuximab for the first-line treatment of metastatic colorectal cancer. *NICE Technology Appraisal Guidance 176, 2009.*http://www.nice.org.uk/nicemedia/pdf/TA176Guidance.pdf. Accessed February 2010

NHS National Institute for Health and Clinical Excellence (NICE). Radiofrequency ablation for colorectal liver metastases. *Interventional Procedure Guidance 327, 2009.*http://www.nice.org.uk/nicemedia/pdf/IPG327FullGuidance.pdf. Accessed February 2010

Nordlinger B, Van Cutsem E, Gruenberger T *et al.* Combination of surgery and chemotherapy and the role of targeted agents in the treatment of patients with colorectal liver metastases: recommendations from an expert panel. *Ann Oncol* 2009; **20**: 985–92.

Nordlinger B, Van Cutsem E, Rougier P *et al.* Does chemotherapy prior to liver resection increase the potential for cure in patients with metastatic colorectal cancer? A report from the European Colorectal Metastases Treatment Group. *Eur J Cancer* 2007; **43**: 2037–45.

Otchy D, Hyman NH, Simmang C *et al.* Practice parameters for colon cancer. *Dis Colon Rectum* 2004; **47**: 1269–84.

Papamichael D, Audisio R, Horiot JC *et al.* Treatment of the elderly colorectal cancer patient: SIOG expert recommendations. *Ann Oncol* 2009; **20**: 5–16.

Porschen R, Sauer R. Interdisciplinary guideline conference ‘Colorectal carcinoma: prevention, diagnosis and treatment 2004’. Subject group VI: Adjuvant and neoadjuvant therapy. *Coloproctology* 2005; **27**: 375–82.

Schmiegel W, Pox C, Adler G *et al.* S3-Guidelines colorectal cancer 2004. *Dtsch Med Wochenschr* 2005; **130**(Suppl. 1): S5–53.

Scottish Intercollegiate Guidelines Network (SIGN). *Management of Colorectal Cancer. A National Clinical Guideline. SIGN Publication no. 67, 2003. Scottish Intercollegiate Guidelines Network (SIGN), Edinburgh (Scotland).*http://www.sign.ac.uk/guidelines/fulltext/67. Accessed February 2010

Society for Surgery of the Alimentary Tract (SSAT). *Surgery for Hepatic Colorectal Metastases, 2004. Society for Surgery of the Alimentary Tract (SSAT), Manchester (MA).*http://www.guideline.gov/summary/summary.aspx?doc_id=5699&nbr=003837&string=colorectal. Accessed February 2010

Tveit KM, Kataja VV. ESMO minimum clinical recommendations for diagnosis, treatment and follow-up of rectal cancer. *Ann Oncol* 2005; **16**(Suppl. 1): i20–1.

Van Cutsem EJD, Oliveira J. Advanced colorectal cancer: ESMO clinical recommendations for diagnosis, treatment and follow-up. *Ann Oncol* 2008; **19**(Suppl. 2): ii33–4.

Van Cutsem E, Oliveira J. Advanced colorectal cancer: ESMO clinical recommendations for diagnosis, treatment and follow-up. *Ann Oncol* 2009; **20**(Suppl. 4): iv61–3.

Van Cutsem E, Nordlinger B, Adam R *et al.* Towards a pan-European consensus on the treatment of patients with colorectal liver metastases. *Eur J Cancer* 2006; **42**: 2212–21.

Van Cutsem EJD, Oliveira J, Kataja VV. ESMO minimum clinical recommendations for diagnosis, treatment and follow-up of advanced colorectal cancer. *Ann Oncol* 2005; **16**(Suppl. 1): i18–19.

Welch S, the Gastrointestinal Cancer Disease Site Group. *The Role of Bevacizumab (Avastin®) Combined with Chemotherapy in the Treatment of Patients with Advanced Colorectal Cancer: Guideline Recommendations, 2008.*http://www.cancercare.on.ca/pdf/pebc2-25s.pdf. Accessed February 2010

Wilke H. An international, multidisciplinary approach to the management of advanced colorectal cancer. *Anticancer Drugs* 1997; **8**(Suppl. 2): S27–31.
